# The influence of self-efficacy on career maturity in college students: mediating the moderation of creativity tendency and achievement motivation

**DOI:** 10.3389/fpsyg.2025.1585195

**Published:** 2025-11-05

**Authors:** Yuying Tong, Ming Zhong, Jiarun Yang, Xiaoxuan Liu, Daiwa Yang, Xueying Zhao, Yixuan Hou

**Affiliations:** ^1^College of Innovation and Entrepreneurship Education, Heilongjiang University, Harbin, Heilongjiang, China; ^2^Harbin University, Harbin, Heilongjiang, China

**Keywords:** college students, self-efficacy, career maturity, creativity tendency, achievement motivation

## Abstract

**Objective:**

This study explores the relationship between self-efficacy and career maturity among college students, while investigating the mediating role of creativity tendency and achievement motivation.

**Methods:**

A survey was conducted on 950 college students using the Self-Efficacy Scale, Career Maturity Scale, Creativity Tendency and Achievement Motivation Scale.

**Results:**

Self-efficacy significantly and positively predicts career maturity. Creativity tendency has a mediating effect between self-efficacy and career maturity among undergraduate students. The interaction between self-efficacy and achievement motivation significantly predicts creativity tendency, while the interaction between creativity tendency and achievement motivation significantly predicts career maturity.

**Conclusion:**

Creativity tendency partially mediates the relationship between self-efficacy and career maturity. Achievement motivation moderates both the initial and later stages of the mediating pathway as “self-efficacy → creativity propensity → career maturity.”

## 1 Instruction

There is a certain urgency in employment situation for college students. Exploring the internal resources of college students, enhancing their career awareness, exploring their career interests, and developing their occupational ability, guiding them to actively improve their employ ability, plays an important role in solving the employment dilemma of college students. Career maturity refers to the degree of psychological preparation an individual possesses to complete career development at one’s appropriate age. The level of preparation reflects 38 the capacity of their ability to choose a career. Super (1955) defined career maturity as the position reached by an individual during the continuous career development process from the exploration stage to the decline stage. Crites (1973) expanded the concept of career maturity and proposed a relatively systematic and mature theory. He emphasized that career maturity reflects an individual’s ability to make career choices, including the knowledge, attitudes, and abilities they possess when making career choices and career planning, as well as the degree of self-awareness. College students are in the exploratory stage of career development, during which the primary goal is to understand themselves and their careers. At this stage individuals should acquire relevant information corresponding to their career development stage, and based on this, make career decisions that are suitable for themselves, and make preliminary career attempts and determine their career.

Previous studies on the influencing factors of career maturity have mostly focused on external factors such as individual demographic factors, family, and society, For example, Hasan (2006) reported that gender has a significant impact on career maturity. Lim and You (2019) categorized parental support for their children’s education into academic support, psychological support, and cultural activity experience support. The study found that these three types of support significantly influence career maturity through the mediating effect of self-esteem. Parental support enhances children’s self-esteem, which in turn affects their career maturity. Im and Kim (2019) found that students who actively participate in class activities and report higher satisfaction with interpersonal relationships exhibit greater career maturity. Choi (2021) suggested that students who perceive high levels of social support have higher career maturity. When students have a better evaluation of the social environment and feel more emotional care in interpersonal interactions, they are more likely to develop stronger self-efficacy and confidence in career decision-making, thereby demonstrating higher career maturity. There are also studies focusing on the impact of psychological factors – such as personality, psychological resilience, and self-efficacy on career maturity (Rahim et al., 2021). However, the specific mechanisms through which psychological factors affect career maturity—particularly the role of self-efficacy and the moderating effects of other variables—remain underexplored. For example, the study found that self-efficacy has a positive impact on career maturity and a significantly enhances college students’ adaptability (Rahim et al., 2021). It plays an important role in future career planning and career choices; however, the specific influencing mechanisms require further investigation. This study primarily investigates the impact mechanism of self-efficacy on career maturity, as well as the roles played by creativity tendency and achievement motivation in enhancing career maturity. It offer theoretical insights and practical implications for further improving the career maturity of college students and enhancing their employment competitiveness.

### 1.1 Relationship between self-efficacy and career maturity

Self-efficacy refers to an individual’s level of belief in their ability to achieve specific domain goals, that is, their perception or belief in their ability to adopt adaptive behaviors when facing challenges in the environment. If a person believes they are capable of the task, they will take action to achieve the goal, and it is a core factor in the individual’s self belief system. Self-efficacy can be divided into general efficacy and specific efficacy. General self-efficacy mainly indirectly affects behavior through specific efficacy. General self-efficacy refers to a person’s self-assessment of successful completion of tasks in the face of various challenges, which is a relatively stable view of the recognition of one’s own ability (Liu et al., 2023). The career decision-making self-efficacy is considered to be the level of confidence of individuals to complete various tasks in the process of career selection. It covers the dimensions of self-evaluation level, career goal selection, future development planning and professional ability cultivation, and is a clearer general sense of self-efficacy (Salim et al., 2023). In fact, many studies have explored the role of self-efficacy in the development of career maturity.

On the one hand, general self-efficacy plays a role in career maturity through career decision-making self-efficacy. For example, a study collected data from 200 college students and analyzed it, and concluded that career decision-making self-efficacy is positively correlated with career maturity (Mohan and Sahu, 2019). That is, when students receive training in career decision-making self-efficacy, the higher their career maturity, and they will choose a suitable career based on their own strengths. The study found a significant relationship between career maturity, career decision-making self-efficacy, parental support, and self-concept (Abdinoor et al., 2019). Hidayat et al. (2019) study found that a positive self-concept is beneficial for improving career maturity.

On the other hand, self-efficacy directly affects career maturity. As suggests, self-efficacy is an important factor influencing a person’s career exploration (Savickas et al., 2018). There are studies indicate that failure in making career planning is related to career immaturity, l career information as well as low self-efficacy (Talib et al., 2010).

Singh and Shukla (2021) used 792 students as a research sample and found a positive correlation between students’ self-efficacy and career maturity from dimensions such as self-evaluation, career information, career choice, career planning, and problem-solving. Social intelligence of college students partially mediates the relationship between self-efficacy and career maturity. The higher the level of self-efficacy of college students, the better they can adapt to the social environment, get along well with others, and have higher career maturity (Wen et al., 2020). Based on this, this study proposes hypothesis 1: College students’ self-efficacy can positively predict their career maturity.

### 1.2 The mediating role of creativity tendency in self-efficacy and career maturity

Self-efficacy primarily affects creative tendencies, and previous studies have explored the role of creative self-efficacy in fostering creativity. Students’ creative self-efficacy in science and mathematics significantly predicted teachers’ evaluation of students’ creativity in these subjects. College students’ creative self-efficacy significantly predicted their trait curiosity, which was measured by their tendency to seek new experiences and their acceptance of unpredictability. It was also positively associated with self-reported originality scores and scores on creative thinking and divergent thinking tests. Furthermore, enhancing creative self-efficacy may strengthen individuals’ creative tendencies (Karwowski, 2012). Creative self-efficacy has a significant positive predictive effect on individual creativity tendencies (Karimi et al., 2021). Huang et al. (2020) reported that the creative self-efficacy of eighth grade students is an important predictor of their creative performance in engineering design. They believe that when individuals are interested and confident in creative tasks/activities, they will engage in deeper information processing and exhibit higher perseverance and willpower in creative activities, resulting in more creative ideas and greater creativity (Chen et al., 2021). Parental support has a significant positive and indirect impact on creativity through the mediating effect of creative self-efficacy (for task-based creativity) or creative interests and self-efficacy (for self-rated creativity), while individuals with high levels of creativity have better divergent and convergent thinking, exhibiting more independent, persistent, confident, and adventurous characteristics. Creative thinking has a positive effect on students’ career decision-making self-efficacy. People with creativity in the workplace report higher job satisfaction and subjective career success rates (Clark and Arnold, 2008). In the digital age, the working environment is constantly changing. The career development of college students has changed from a traditional road to a new track of dynamic change. It is very important to pay attention to the relationship between creative self-efficacy and promoting professional behavior. They believe that having creative self-efficacy in the workplace can help college students make more proactive professional behaviors in the new working environment, which is conducive to the development of their professional maturity (Gerçek and Özveren, 2025). Individuals who were generative in their work reported greater job satisfaction and subjective career success, and creative thinking helps college students develop confidence in making career decisions and plans, which is beneficial for their future career development and maturity (Fan, 2016). Therefore, this article hypothesizes that creativity plays a mediating role between self-efficacy and career maturity.

### 1.3 The moderating effect of achievement motivation

Achievement motivation refers to the intrinsic drive and psychological inclination to strive for excellence in order to achieve higher goals, and helps to develop individuals’ thinking, enhance their creativity, and has a positive effect on realizing their potential and intrinsic value. Individual achievement motivation is closely related to creativity tendency (Ma et al., 2024). High achievement motivation may prompt individuals to accept challenges, overcome difficulties, and complete high-quality creative activities. Under the goal orientation of creative activities, the motivation to pursue success promotes the initiation and persistence of creative behavior. Students with high self-efficacy typically possess strong achievement motivation. They tend to select challenging goals and tasks, show a willingness to take risks, and display curiosity and imagination, which enable them to break conventional thinking. They tend to have high creativity, and to be confident in their ability to propose effective creative strategies (Green et al., 2024). Even if they may face difficulties or setbacks, they will explore and overcome fatigue to achieve optimal results and unleash their creative tendencies. The level of creativity also affects career maturity. When facing problems or tasks, their strong desire to pursue success emphasizes the development factors in their careers. College students who pursue success motivation have clear goals, accurate positioning, and a deeper understanding of themselves and their positions. A strong sense of creative self-efficacy is imperative for preparing students for the creative expectations of their future careers and to participate fully in today’s high-pressure society. People with the motivation to avoid failure always wrap themselves in a small world, afraid to try, and fail to develop their professional confidence and abilities due to the fear of failure and being ridiculed (Green et al., 2024).

**Figure 1:**
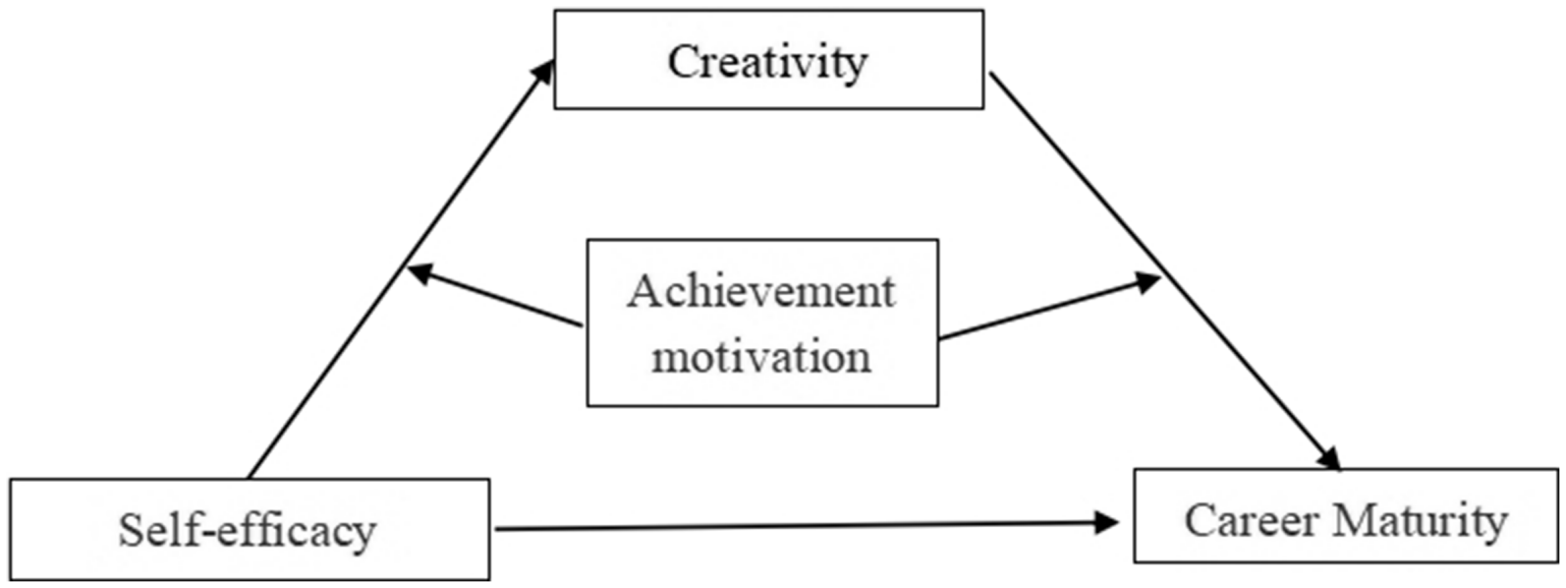
A moderated mediating model.

In summary, based on previous research, this study aims to develop a conceptual framework for the career maturity, self-efficacy, achievement motivation, and creativity tendency of college students, and to analyze the relationship among these factors. A moderated mediation model (see Figure 1) is constructed to explore the impact of self-efficacy on career maturity, as well as the impact mechanism of creativity tendency and achievement motivation. The research results not only further clarify the relationship and mechanism of self-efficacy on career maturity, but also the role of creativity tendency and achievement motivation in this process. These results offer a new theoretical perspective for the impact mechanism of career maturity, analyze the influencing factors of career maturity, and propose corresponding educational strategies to improve the career maturity of college students. Enhancing career maturity can offer both theoretical guidance and empirical evidence to support college students’ career decision-making. This process helps students strengthen their career maturity, make informed career planning choices, and ultimately maximize their employment success.

## 2 Participants and methods

### 2.1 Participants

This study adopts the online random sampling method. The Participants were college students from 11 universities. Demographic factors such as grade level, subject major, and family background are considered to improve the universality of research results. Participants voluntarily click on the link to fill in the questionnaire, inform the purpose of the questionnaire before filling in the questionnaire, and can withdraw at any time in the process to fully ensure the privacy and rights of the respondents. A total of 1,015 questionnaires were sent out through “Questionnaire Star,” and 950 valid questionnaires were obtained, with an effective questionnaire rate of 93.59%. There were 358 males (37.7%) and 592 females (62.3%). This study was approved by the Ethics Committee of Heilongjiang University, and the investigation method complied with relevant guidelines, laws and regulations. All students provided informed consent forms.

### 2.2 Materials

#### 2.2.1 General self-efficacy scale, GSES

The General Self Efficacy Scale (GSES) developed by Zhang and Schwarze includes 10 items (Skliarova et al., 2023). The scale is 4 point Likert type scale: “completely incorrect” (1), “slightly correct” (2), “mostly correct” (3), “completely correct” (4).” The higher the average score, the higher the individual’s self-efficacy. The Cronbach’s alpha for the scale was 0.942 in this study.

#### 2.2.2 Career maturity scale, CMS

In the scale there are statements which measure attitudes and behaviors related to career choice (Birola and Kiralp, 2010). The scale is 5 point Likert type scale: “not at all like me” (1), “not much like me” (2), “somewhat like me” (3), “like me” (4), “very much like me” (5). The student who reached the raw score of 143 shows that the student reached the career maturity level that is expected from him. The Cronbach alpha for the scale was 0.964 in this study.

#### 2.2.3 Williams creativity assessment packet, CAP

CAP, compiled by Cooper (1991) and revised by Liu et al. (2016), this questionnaire consists of 50 items, including 4 dimensions: adventure, curiosity, imagination, and challenge; The scale is 3 point Likert type scale: “completely disagree” (1), “partially agree” (2), “completely agree” (3). The higher the score, the more obvious the tendency toward creativity. The Cronbach’s alpha for the questionnaire was 0.964 in the study.

#### 2.2.4 The achievement motive scale (AMS)

The Achievement Motive Scale (AMS) is a revised translation by Li et al. (2023), which includes 30 items. The scale is 5 point Likert type scale: “being completely inconsistent” (1), “being somewhat inconsistent” (2), “being uncertain” (3), “being somewhat consistent”(4), “being very consistent” (5). The higher the score, the stronger the motivation. The Cronbach’s alpha for the scale was 0.951 in this study.

### 2.3 Data analysis

SPSS 27.0 statistical software were used for the descriptive and correlational analysis among self-efficacy, career maturity, creativity tendency and achievement motivation. The Model of SPSS’s Process macro (Hayes, 2022) was employed to test the moderated mediating effect of creativity tendency and achievement motivation on the relationship between self-efficacy, career maturity. The significance level was set to 0.05 (*p* < 0.05).

## 3 Results

### 3.1 Common method bias

This study is based on a questionnaire survey and may have common method bias. Therefore, Harman single factor test (Aguirre-Urreta and Hu, 2019) was used to test for common method bias. The non-rotated factor analysis results showed that there were 17 factors with eigenvalues greater than 1. The total variation of the first factor was 29.815%, which was below the critical value of 40%, indicating that there was no serious common method bias in the study.

### 3.2 Describe statistics and correlation analysis

Pearson correlation analysis was conducted on college students’ self-efficacy, career maturity, creativity tendency and achievement motivation, results are given in Table 1. There was significant positive correlation between career maturity and self-efficacy, career maturity and creativity tendency, and career maturity and achievement motivation (*Ps*<0.05). There also was a significant positive correlation between self-efficacy and creativity tendency (*p* < 0.05), no significant relationship between self-efficacy and achievement motivation (*p* > 0.05), and significant negative correlation between creativity tendency and achievement motivation (*p* < 0.05).

**Table 1:** Relevant descriptive statistical analysis of career maturity, self-efficacy, creativity tendency and achievement motivation.

Variable	*M* ± SD	Career maturity	Self-efficacy	Creativity tendency
Career maturity	121.31 ± 21.66			
Self-efficacy	21.33 ± 6.77	0.427^**^		
Creativity tendency	88.80 ± 14.78	0.412^**^	0.522^**^	
Achievement motive	1.95 ± 4.02	0.210^**^	0.026	−0.082^*^

^*^P < 0.05, ^**^p < 0.01.

### 3.3 The relationship between self-efficacy and career maturity: a moderated mediation model test

According to Hayes (2022) suggestion, examine the mediating role of creativity tendency and the moderating role of achievement motivation. Use the Process Procedure for SPSS Version 4.1 model to test for moderated mediation effects. Equation 1: Tests the moderating effect of achievement motivation on the relationship between self-efficacy and career maturity; Equation 2 tests the moderating effect of achievement motivation on the relationship between self-efficacy and creativity tendency; Equation 3 tests the moderating effect of achievement motivation on the relationship between creativity tendency and career maturity. All variables are standardized, and the parameter estimation results of each equation are shown in Table 2.

**Table 2:** The relationship between self-efficacy and career maturity: a moderated mediation effect test.

Predictor variable	Equation1: Career maturity				Equation2: Creativity tendency				Equation3: Career maturity			
	*β*	SE	*t*	95%CI	*β*	SE	*t*	95%CI	*β*	SE	*t*	95%CI
Constant	121.42	0.60	202.49	[120.24, 122.59]	61.85	1.41	43.90	[59.08, 64.61]	99.49	2.49	39.88	[94.59, 104.38]
Self-efficacy (X)	0.93	0.11	8.51^***^	[0.72, 1.15]	1.31	0.06	20.55^***^	[1.19, 1.44]	1.03	0.11	9.28^***^	[0.81, 1.25]
Achievement motivation (W)	−1.26	0.18	−6.91^***^	[−1.62, −0.90]	1.54	0.29	5.37^***^	[0.98, 2.10]	−1.28	0.19	−6.60^***^	[−1.66, −0.90]
X*W	0.01	0.03	0.31	[−0.05, 0.07]	−0.10	0.02	−6.86^***^	[−0.12, −0.07]				
Creativity tendency (M)									0.33	0.05	6.33^***^	[0.23, 0.43]
M*W									0.02	0.01	2.84	[0.01, 0.04]
*R^2^*			0.31				0.31				0.29	
*F*			119.06^***^				144.32^***^				77.92^***^	

^***^P < 0.001.

The results showed that self-efficacy had a significant positive predictive effect on career maturity (*β* = 0.93, *t* = 8.76, *p* < 0.001, 95% CI [0.72, 1.14]). Therefore, it can be concluded that hypothesis 1 has been confirmed, and self-efficacy has a significant positive predictive effect on creativity tendency (*β* = 1.31, *t* = 20.55, *p* < 0.001, 95%CI[1.19, 1.44]), and creativity tendency has a significant positive predictive effect on career maturity (*β* = 0.33, *t* = 6.33, *p* < 0.001, 95%CI [0.23, 0.43]), Indirect effect (s) of X on Y (Effect = 0.44, Boot SE = 0.07, 95%CI[0.31, 0.57]), excluding 0. Therefore, hypothesis 2 has been confirmed. Confirming that creativity tendency plays a mediating role between self-efficacy and career maturity; The interaction between self-efficacy and achievement motivation has no significant predictive effect on career maturity (*β* = 0.01, *t* = 0.31, *p* < 0.001, 95%CI[−0.05, 0.07]); The interaction between self-efficacy and achievement motivation has a significant predictive effect on creativity tendency (*β* = −0.10, *t* = −3.68, *p* < 0.001, 95%CI[−0.15, −0.05]); The interaction between creativity tendency and achievement motivation has a significant predictive effect on career maturity (*β* = 0.02, *t* = 2.84, *p* < 0.001, 95%CI[0.01, 0.04]). Therefore, hypothesis 3 has been confirmed, and achievement motivation moderates the effect of self-efficacy on college students’ career maturity via creative tendency, influencing both the initial and later stages of the pathway “self-efficacy → creative tendency → career maturity.”

**Figure 2:**
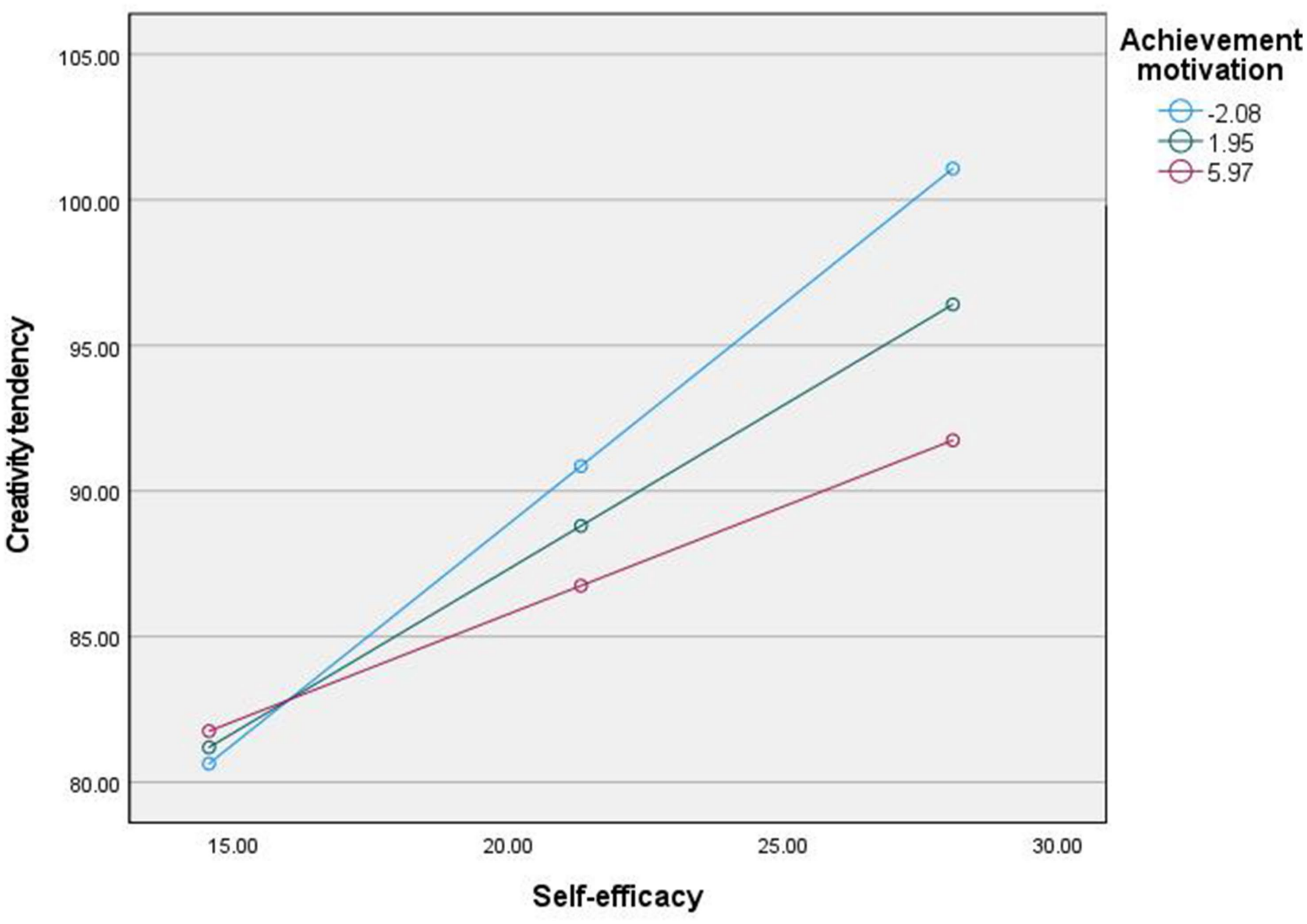
The moderating effect of achievement motivation in the first half.

**Figure 3:**
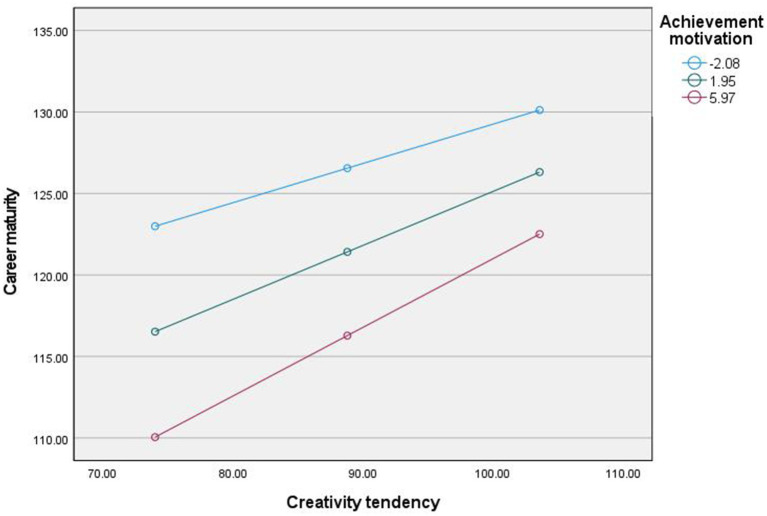
The moderating effect of achievement motivation in the second half.

Using a simple slope test to further analyze how achievement motivation regulates the relationship between self-efficacy and career maturity, achievement motivation is divided into high group (M + 1SD) and low group (M − 1SD) according to M ± 1SD. As shown in Figures 2, 3, as well as Tables 3, 4, when the level of achievement motivation is low (M − 1SD), the positive predictive effect of creativity tendency on career maturity is significant; When the level of achievement motivation is moderate (M), the positive predictive effect of creativity tendency on career maturity is significant; When the level of achievement motivation is high (M + 1SD), the positive predictive effect of creativity tendency on career maturity is significant. The results show that as the level of achievement motivation increases, the predictive effect of creativity tendency on career maturity gradually increases.

**Table 3:** Mediating effect of self-efficacy on creativity tendency at different levels of motivation.

Index	Achievement motivation	Effect	Boot SE	*t*	*P*	LLCI	ULCI
Creativity tendency	Mean − SD	1.51	0.08	18.96	0.000	1.35	1.67
	Mean	1.12	0.06	19.09	0.000	1.01	1.24
	Mean + SD	0.74	0.08	8.87	0.000	0.58	0.90

**Table 4:** Mediating effect of creativity tendency on career maturity at different levels of motivation.

Index	Achievement motivation	Effect	Boot SE	*t*	*P*	LLCI	ULCI
Career maturity	Mean − SD	0.24	0.06	4.01	0.000	0.13	0.36
	Mean	0.34	0.05	6.87	0.000	0.24	0.43
	Mean + SD	0.43	0.06	7.52	0.000	0.32	0.54

## 4 Discussion

### 4.1 The relationship between self-efficacy and career maturity

This study tests correlational relationships suggested by the model of individual differences in career maturity, which carry substantial theoretical and practical significance. It explores the relationship between self-efficacy scales and career maturity, and the results show that self-efficacy can positively predict the career maturity of college students. Hypothesis 1 has been validated, which is consistent with previous research (Green et al., 2024). According to The Social Cognitive Theory (SCT), self-efficacy represents the core mechanism of individual agency. It influences not only affects individuals’ effort and persistence, but also influences their choices of activities and behavioral environments (Bandura, 1986). College students are in a critical period of career cognition and career choice, and high levels of career-related self-efficacy has a significantly impact on career maturity (Lee et al., 2020). Some research results have shown that Parental attachment through self-efficacy has an influence on career maturity (Qudsiyah et al., 2018). A study found that self-efficacy has a positive impact on career maturity and a significant impact on improving college students’ adaptability, playing an important role in future career planning and choices (Rahim et al., 2021). Subsequent analysis of the mediating role of career decision-making self-efficacy indicates that it exerts a partial mediating effect (Duru, 2022). The dual mediating effect of career identity and career decision-making self-efficacy on the relationship between perceived social support and career maturity in primary school students, where the improvement of self-efficacy contributes to the enhancement of individual career maturity.

### 4.2 The mediating role of creativity tendency

The results of this study indicate that self-efficacy not only directly affects career maturity, but also indirectly affects it through creative tendencies, verifying hypothesis 2. According to Karwowski and Kaufman (2017), CSE influences a individuals’ creativity and their propensity to engage in creative performance or efforts to address creative challenges. Individuals with high self-efficacy possess provides the cognitive resources and motivation to persist in creative activities cognitive resources and motivation to persist in creative activities (Valquaresma et al., 2022), which is beneficial for fully unleashing one’s creativity. Research indicates that CSE not only predicts but also related to creative performance (Chang et al., 2016), creative imagination (Yang et al., 2020), creative potential (Puente-Diaz et al., 2020), and creative problem-solving (Royston and Reiter-Palmon, 2019) among college/university students. CSE has been shown to be associated with creative production (Smith, 2022). Individuals with high self-efficacy are more willing to explore external things, more willing to explore themselves, more willing to spend time researching and pondering new things, curious about the outside world, imaginative, more adventurous, and willing to accept challenges. Creative individuals possess a high degree of self-evaluation, are able to accurately accept various ideas and experiences, have stronger self-efficacy and confidence in career decision-making, and thus demonstrate higher career maturity. It can be seen that individuals with high self-efficacy have stronger creativity and thus possess better career maturity.

### 4.3 The moderating effect of achievement motivation

The results of this study indicate that achievement motivation moderates the mediating role of creativity tendency in both the initial and later stages of the mediating pathways between self-efficacy and career maturity. There is no significant direct interaction between self-efficacy and motivation for achievement in terms of professional maturity. It may be because the interaction between these independent variables has little impact on the dependent variables, and the main effect can be mainly focused on. It may also be because some results are underestimated by the traditional effect quantity criteria, and the non-remarkable results may actually reveal the limitations of the theory in different situations. Therefore, this article mainly interprets from the main effect. Among individuals with the motivation to avoid failure, self-efficacy has a mediating effect through the inclination of creativity toward career maturity. For individuals with the motivation to pursue success, self-efficacy has a greater mediating effect through the inclination of creativity toward career maturity. This is consistent with previous research that the achievement motivation of high school students can affect their career maturity (Chen and Han, 2022). This study used college students as participants and obtained similar results. According to the theory of social achievement motivation, achievement motivation is the person’s tendency to pursue specific goals. This finding is in the line with Santos et al. (2010) and Heydari (2010), achievement motivation has a significant positive relation with creativity (Ghasemi et al., 2011). When individuals hold higher expectations for their future careers or lives, coupled with stronger self-efficacy and greater confidence, their intrinsic motivation to achieve success is enhanced, which in turn promotes greater creativity. In this process, they will constantly adjust their goals and strive to seek innovative solutions, which in turn promoting individuals to achieve higher career maturity. From the perspective of education, artificial intelligence is changing the traditional Chinese teaching methods, guiding students to make the correct motivation attribution of achievement, and improving the enthusiasm and participation of learners. This is an important way to promote the success of learners and an important means to optimize practical learning results in the digital education environment. This study emphasizes the value of achievement motivation theory and provides actionable insights for creating supportive and technologically enhanced vocational maturity training (Zhao, 2025). So it is not difficult to understand that achievement motivation plays a good moderating role between self-efficacy and creativity tendency.

## 5 Limitations

First of all, a key limitation of this study is its reliance on cross-sectional data, which precludes causal inferences. The analysis of the existing research results in the field needs to be deepened vertically. While our findings suggest associations between self-efficacy and career maturity, alternative causal models (e.g., career maturity influencing self-efficacy) remain plausible. Future research should employ longitudinal designs to better establish temporal precedence and causal relationships. Additionally, it should committed to combine forward-looking and realistic, expand sample sizes, and refine experimental designs to assess the long-term impact of self-efficacy on career maturity. Secondly, this article examines the level of achievement motivation, but due to the limitations of research resources, it may not cover all research dimensions or samples. But it is important for future studies to distinguish motivation to succeed and to avoid failure. Thirdly, this study only aims to explore the impact of self-efficacy on the career maturity of college students within the context of Chinese central culture, and does not address the relationship in other social and cultural contexts. Future research should incorporate cross-cultural studies to assess the theoretical robustness across different cultural contexts.

## 6 Recommendations

### 6.1 Encourage students’ creative development

As an important influencing factor in the future career development of college students, career maturity is of great value to the cultivation of college students’ individual development ability and future career planning and decision-making. Therefore, schools and teachers should further strengthen the guidance of college students’ professional concept and sense of self-efficacy, adopt educational practice to maintain individual positive emotions in professional practice, and improve the professional self-identity level of college students. At the same time, the relevant educational part of the school needs to constantly update the educational and teaching practice resources, enrich the content of educational practice, strengthen in-depth communication on the cultivation of professional ability on the basis of a broad-spectrum curriculum, pay attention to students’ inner career world, stimulate their creativity and creative thinking as much as possible, and enhance core competitiveness, and strengthen the training of professional maturity.

### 6.2 Guide students to form a reasonable motivation for achievement

Simultaneously, schools should encourage college students to cultivate positive achievement motivation, optimally coordinate educational resources, and systematically integrate emotional intelligence education into the overall educational process. Additionally, students should carefully assess the alignment between their target occupations and academic majors, taking into account professional characteristics, individual differences, and external social support, in order to promote healthy development of career maturity.

## 7 Conclusion

In conclusion, creativity tendency partially mediates the relationship between self-efficacy and career maturity. Achievement motivation moderates both the initial and later stages of the mediating pathway as “self-efficacy → creativity propensity → career maturity.”

## Data Availability

The original contributions presented in the study are included in the article/supplementary material, further inquiries can be directed to the corresponding author.
